# Chronic Arterial Degeneration

**Published:** 1906-12-29

**Authors:** 


### CHRONIC ARTERIAL DEGENERATION.

The literature of arterial degeneration is immense and not a little baffling on account of the very varied classifications of the disease which have commended themselves to different authorities. There is, in point of fact, no rigid differentiation of types as regards the post-mortem evidences of lesion in the arteries. Microscopic examination indicates that the tunica intima is the first of the three arterial coats to evince signs of degeneration in the majority of cases, although the other coats are commonly involved either at the same time or later. A general consideration of the morbid histology of degenerate arteries lends justification to the conception of chronic degenerative arteritis, wherever it occurs, as commencing in a fibro-cellular deposit in the sub-endothelial connective tissues of the tunica intima. The ultimate fate of this deposit, in conjunction with the distribution of the lesion about the arterial system, will dictate the type of the degeneration. Thus in a great number of cases this sub-endothelial deposit tends to undergo a fatty disintegration, as a result of which there is produced a raised yellow plaque upon the inner wall of the vessel, the well-known atheromatous plaque. As the disintegration becomes more pronounced the intimal deposit becomes more liquid, constituting an atheromatous abscess; and finally, when by erosion of the covering endothelium the abscess ruptures into the lumen of the vessel, its semi-fluid contents are discharged into the circulation and there remains an atheromatous ulcer the base of which is formed by the tunica media. ἀθήρ signifies porridge or gruel, and the name atheroma has been given to this degeneration to indicate the gruel-like constituents of the atheromatous abscess.

In consequence, the term atheroma should be confined to those lesions which are marked by a fatty degeneration of the intimal deposit. So defined, atheroma is found to affect the larger arteries by preference. The aorta is thus by far the most frequent site of its assault, although no arteries, however small, seem to be immune to this degeneration. As a final sequence of the atheromatous process it is common to find a deposit of calcareous salts in the area of the ulcer, a sequence which leads not infrequently to the transformation of the affected area into a calcareous plate.

But although atheroma can be thus pathologically defined with strictness, it does not as a rule monopolise the arterial lesions in any given instance of arterial degeneration. The intimal deposit which has been assumed to be present in all cases may undergo other degenerations besides that resulting in a fatty débris. Of these the two commonest are a fibrous and a hyaline variety. These two forms affect by preference the smaller arteries, and especially the capillary arterioles of the kidney. The process is commonly a widespread one, and constitutes the condition to which Gull and Sutton gave the name of arterio-capillary fibrosis. When the degeneration is fibrous, a cross-section of the arteriole in question may show no trace of an elastic membrane or of an intimal endothelium. The lumen of the vessel is invaded to a variable degree by concentric bands of fibrous tissue which have replaced the tunica intima and merge insensibly with the more or less fibrosed tunica media. This is a common appearance in sections of the red granular or arteriosclerotic kidney. The hyaline variety of the degeneration is marked by a deep layer of hyaline material, the site of which is proved to be sub-endothelial by the fact that the endothelium may often be seen to be intact upon its inner surface, while the fenestrated elastic membrane defines it on the outer side. The hyaline material is scantily cellular, but shows no tendency to liquefy. It marks a variety of degeneration which is seen in its most typical form in arteries lying in the vicinity of gummatous infiltrations, and occasionally as a diffuse arterial affection of the syphilitic type apart from any local gummatous affection. Ziegler considers that there is nothing specific about this appearance, although some authorities hold it to be peculiarly a syphilitic lesion, and call it "syphilitic endarteritis or endarteritis obliterans."

We may therefore conceive of chronic arteritis as comprising three main histological groups, atheromatous endarteritis, fibrous endarteritis, and hyaline endarteritis, including in the latter category the syphilitic cases, whether definitely gummatous or not. It is idle to consider these varieties as separate entities, for they may all be found in different regions of the same arterial system, and the whole may fairly be embraced under the generic title arteriosclerosis. The determining factor in the causation of arteriosclerosis in any given case is seldom to be rigidly identified, for a great variety of causes appear to be competent to bring about identical results. It is possible, however, to distinguish two well-defined elements at work. There is, in the first place, the hereditary factor. It is past question that the endowment of arterial resistance varies within wide limits: that some individuals evince an innate tendency to arterial degeneration; and that such a tendency is transmissible to offspring. For the rest, arterial degeneration is evidence of intoxication, of the slow poisoning of the artery by some harmful body circulating with the blood. In a few cases the nature of the poison may be defined with sufficient assurance to justify presumption of some similar agent in cases of more obscure causation. For instance, we cannot doubt that the circulation of salts of lead in the blood exercises a peculiarly harmful effect upon the arterial walls. Syphilis, similarly, is capable of acting as a direct poison to the tunica intima, and this quite apart from local onslaughts of the parasite. We must assume, therefore, the presence in such cases of some slowly acting diffusible poison. Lastly, alcohol is a well-recognised and potent destroyer of arteries. We have in these three accepted enemies of arterial health representatives of three widely different chemical groups, one organic, one inorganic, and one parasitic. Yet they appear equally capable of producing a chronic sclerosis of arteries, and warrant us in assuming some comparable factor of destruction in cases which appear more mysterious. We may therefore conclude the ætiological consideration of the subject by the dogma that chronic arteritis depends upon a circulating poison, and that its incidence and histological variety will be determined by the nature and dose of the poison and by the individual's endowment of arterial resistance to such poison. Such an expression of opinion shares the limitations of all dogmas, but it yields a justifiable working hypothesis.

The results of arteriosclerosis (and, in consequence, its symptoms) will be dictated by the locality of the lesion more than by any other single factor—that is to say, by the diameter of the vessel at the seat of attack. Where the seat of the lesion is in a large artery, such as the aorta, the results of the lesion are likely to be localised; when, however, the smaller arteries are attacked, the evidences of the disorder are likely to be general as well as local, for the process is then in all probability diffuse. For instance, in the case of aortic sclerosis the lesion is generally atheroma, as we have already said. The explanation of this preference is not determined, but some authorities affirm its dependence upon an endarteritis of the vasa vasorum of the large vessels with consequent anæmic necrosis of the area of vessel-wall dependent upon these nutritive vessels. But whatever the explanation, the fact remains that in the aorta and its large branches atheroma is the predominant lesion. The direct result of such damage is a weakening of the arterial tube at that part of its circuit where the calls upon its resistance and resilience are most pronounced. As a natural consequence of these circumstances, we find in this part of the arterial tree a liability to aneurysmal dilatation far in advance of that shown by any other portion. Thus of 631 cases of aneurysm occurring in the practice of St. Bartholomew's Hospital no fewer than 468 were aortic in situation. When, however, the stress of the degeneration falls upon the smaller vessels, the determining element in the clinical course of the malady is the obliteration of the arterial lumina brought about by the subendothelial deposits mentioned above. This will reflect itself both distally and proximally—that is to say, both upon the tissues supplied by the narrowed artery and upon the heart. To illustrate the former of these two effects we may briefly consider the four situations where arterial disease of this type is most in evidence—namely, the brain, the heart, the kidneys, and the extremities. In the case of the brain, as elsewhere, a gradual narrowing of its arteries will result, of course, in a relative anæmia of the brain substance. This will show itself symptomatically in temporary lapses of memory, confusion of thought, vertigo, and the like. Further, the roughening of the arterial lumen produced by the endarterial excrescences will predispose to the clotting of blood in the vessels, in which case the result will be a thrombotic softening of the region of brain-substance thus deprived of nutrition. The symptoms will then indicate the seat of the necrosis, either by monoplegia, hemiplegia, aphasia, or, when the silent areas of the brain are affected, other vaguer disturbances of consciousness.

Since the blood-supply of the heart-muscle depends upon the coronary arteries, it is easy to see how readily a diffuse sclerosis of the aorta may involve these arteries at their origins in the sinuses of Valsalva. At other times the coronary vessels themselves are affected in their course, but in either case the result is a relative anæmia of the heart muscle. Such a condition will manifest itself in the case of the heart at an earlier stage than in any other situation in the body, since upon the untiring efficiency of the heart muscle the general welfare of the whole organism directly depends. The defect shows itself in the well-known signals of cardiac distress. Palpitation, breathlessness on slight exertion, cardiac pain, whether præcordial or, as is more often the case, pain in the peripheral distribution of the corresponding spinal segments—that is to say, the arm. Any or all of these may confess the embarrassment of the heart and reflect the slow degeneration of its muscle-fibres through lack of food. Finally, after many months of disability, the unequal contest may be closed by an attack of syncope.

The anæmic atrophy of the kidney which results from the gradual obstruction of its arterioles of supply shows itself histologically in a degree of fibrosis of the kidney tissue which sometimes leaves it with little title to be called a glandular organ. It appears then to the naked eye as a small, red, hard body, with a granular surface closely adherent to its capsule, and often carrying cysts which represent urinary tubules the exit of which has been closed by the cicatricial contraction of newly formed fibrous tissue, and in which urinary secretion has in consequence accumulated. The symptoms associated with this advanced degree of renal destruction are often singularly insignificant. As regards its identification by means of the urine, it may be said that a reduction of the specific gravity may be the only evidence of the lesion, although there is commonly a slight degree of albuminuria. The main evidence of the disease is to be found in the accompanying hypertrophy of the heart and in elevation of arterial tension, to which reference will presently be made.

A precisely analogous sequence of events will, when the arteries to the extremities bear the brunt of the degeneration, lead to evidences of deficient nutrition. This condition finds its most typical expression in the condition known as senile gangrene, which is no more than the result of arterial obliteration due to an obstructive endarteritis, often assisted in its concluding phases by thrombosis. But apart from actual gangrene it is not uncommon to meet with patients evincing in the hardness of their radial arteries a generalised arterio-sclerosis, and who at the same time show, by the blueness of their fingers or toes, the degree of arterial obstruction under which they labour.

We have said that the heart, as the propulsive engine of the circulation, shares in the results of a diffused arterio-sclerosis. Hypertrophy of the heart, and an increase in the tension of the pulse, are almost invariably accompaniments of diffuse arteriosclerosis. We have seen also that the red granular kidney is equally a common accompaniment of the condition. It is no wonder, therefore, that we find medical opinion divided into two camps, one upholding the priority of the kidney lesion, the other that of the arterial disturbance. The matter is still *sub judice* and need not here be debated at length. Given the narrowing of the peripheral arterioles it is easy to comprehend that the driving of an adequate supply of blood through an unnaturally narrow series of tubes postulates increased strength in the heart and a higher tension of the blood in the arteries.

The treatment of arterio-sclerosis must be either preventive or merely symptomatic. It is probable that, in addition to the poisons already mentioned, those which most commonly promote arterial degenerations are certain products of perverted metabolism. It is further certain that these toxic influences are particularly associated with an over-generous diet and a sedentary line of life. These facts indicate sufficiently the direction of prophylactic treatment. As Bacon says, "Discern of the coming on of years, and think not to do the same things still; for age will not be defied." When symptoms appear they depend very often upon the obstruction offered to the failing heart by the co-existent elevation of blood-pressure. Treatment should therefore aim at the reduction of blood-pressure. This may be achieved, and the comfort of the patient immensely increased, by the exhibition of nitroglycerine, by the use of saline purges, and by a reduced consumption of food and drink.

